# A mixed methods evaluation of patient perspectives on the implementation of an electronic health record-integrated patient-reported symptom and needs monitoring program in cancer care

**DOI:** 10.1186/s41687-024-00742-8

**Published:** 2024-07-02

**Authors:** Madison J. Lyleroehr, Kimberly A. Webster, Laura M. Perry, Elijah A. Patten, Jackelyn Cantoral, Justin D. Smith, David Cella, Frank J. Penedo, Sofia F. Garcia

**Affiliations:** 1 Department of Medical Social Sciences, Northwestern University Feinberg School of Medicine, 625 N. Michigan Ave., Suite 2100, Chicago, IL 60611 USA; 2 Robert H. Lurie Comprehensive Cancer Center, Northwestern University, 675 N. St. Clair St. Fl 21 Ste 100, Chicago, IL 60611 USA; 3 Biological Sciences Division, University of Chicago, Chicago, IL 60637 USA; 4 Department of Population Health Sciences, Division of Health System Innovation and Research, Spencer Fox Eccles School of Medicine at the University of Utah, Salt Lake City, UT 84108 USA; 5 Departments of Psychology and Medicine, University of Miami, Coral Gables, FL 33124 USA; 6 Sylvester Comprehensive Cancer Center, Miller School of Medicine, University of Miami, Miami, FL 33136 USA

## Abstract

**Background:**

As cancer centers have increased focus on patient-centered, evidenced-based care, implementing efficient programs that facilitate effective patient-clinician communication remains critical. We implemented an electronic health record-integrated patient-reported symptom and needs monitoring program (‘cPRO’ for *c*ancer *p*atient-*r*eported *o*utcomes). To aid evaluation of cPRO implementation, we asked patients receiving care in one of three geographical regions of an academic healthcare system about their experiences.

**Methods:**

Using a sequential mixed-methods approach, we collected feedback in two waves. *Wave 1* included virtual focus groups and interviews with patients who had completed cPRO. In *Wave 2*, we administered a structured survey to systematically examine *Wave 1* themes. All participants had a diagnosed malignancy and received at least 2 invitations to complete cPRO. We used rapid and traditional qualitative methods to analyze *Wave 1* data and focused on identifying facilitators and barriers to cPRO implementation. *Wave 2* data were analyzed descriptively.

**Results:**

Participants (*n* = 180) were on average 62.9 years old; were majority female, White, non-Hispanic, and married; and represented various cancer types and phases of treatment. *Wave 1* participants (*n* = 37) identified facilitators, including cPRO’s perceived value and favorable usability, and barriers, including confusion about cPRO’s purpose and various considerations for responding. High levels of clinician engagement with, and patient education on, cPRO were described as facilitators while low levels were described as barriers. *Wave 2* (*n* = 143) data demonstrated high endorsement rates of cPRO’s usability on domains such as navigability (91.6%), comprehensibility (98.7%), and relevance (82.4%). *Wave 2* data also indicated low rates of understanding cPRO’s purpose (56.7%), education from care teams about cPRO (22.5%), and discussing results of cPRO with care teams (16.3%).

**Conclusions:**

While patients reported high value and ease of use when completing cPRO, they also reported areas of confusion, emphasizing the importance of patient education on the purpose and use of cPRO and clinician engagement to sustain participation. These results guided successful implementation changes and will inform future improvements.

**Supplementary Information:**

The online version contains supplementary material available at 10.1186/s41687-024-00742-8.

## Background

### Context

Cancer care has shifted toward patient-centeredness, which prioritizes delivering evidence-based, quality care to improve patient outcomes [1, 2]. Accordingly, healthcare organizations are learning how to effectively implement new standards of cancer care, including those that address patient-level outcomes (i.e., patient-reported quality of life, symptoms, treatment satisfaction, and experiences with healthcare systems). Research has demonstrated that patient-centered cancer care can improve multilevel patient outcomes, including survival [3, 4]. 

While advances in cancer screening and therapeutics have made notable impacts on survival, this benefit can be offset by compromises in health-related quality of life (QOL) [5]. Patients commonly receive multimodal treatments with varying toxicities that can complicate symptom management and negatively impact QOL. Literature highlights the prevalence, persistence, and burden of disease- and treatment-related symptoms and the psychosocial sequalae of living with a chronic or life-threatening condition, as well as needs related to practical concerns (e.g., nutritional or financial), which may go untreated without proactive clinical management systems [6–10]. Additional evidence suggests that effective communication between patients and clinicians remains a challenge [11, 12] and that poor communication can adversely impact health and other relevant outcomes [11, 13]. Implementing programs within routine practice to better identify, communicate, and manage patients’ health needs can promote patient-centered care that improves individual outcomes. Prior evaluations of routine symptom monitoring in cancer care have demonstrated multilevel clinical utility and value, and potential to influence meaningful outcomes [3, 4, 14]. 

However, implementing an intervention as a standard of care requires strategic planning and iterative evaluation [15, 16]. Use of implementation science (IS) methods to bridge the research-to-practice translation gap has been shown to augment success [17, 18]. IS offers methodological and evaluative models and frameworks to inform implementation processes, drive adoption and system integration, and assess the effects of implementation efforts, including identifying facilitators and barriers and the strategies required to support uptake and sustained delivery [19]. The goal of IS is to facilitate the uptake of evidence-based practice and research evidence into regular use by practitioners, health systems, and health policymakers [20]. Implementation scientists commonly focus on strategies—methods or techniques used to enhance the uptake, implementation, and sustainment of research evidence [21]—that align with context-specific barriers and facilitators to support uptake [19]. IS goes beyond effectiveness of interventions and health innovations to understand the system processes, resources, and capacities needed to support sustained use of best available research evidence [22]. 

### Preliminary work

To address the need for comprehensive symptom monitoring in cancer care, we previously developed and piloted an electronic health record-integrated patient-reported symptom and needs monitoring program (‘cPRO’ for *c*ancer *p*atient-*r*eported *o*utcomes) within Northwestern Medicine’s (NM) electronic health record (EHR) [23, 24]. cPRO is custom-designed to administer validated patient-reported outcome (PRO) measures that assess key symptoms in oncology (depression, anxiety, fatigue, pain interference, and physical function) from the Patient-Reported Outcomes Measurement Information System (PROMIS®) [25, 26] and a checklist to identify supportive care needs. The automated system releases cPRO assessments 72 h before oncology appointments (limited to once every 30 days) and is completed by patients via the EHR patient portal prior to their visits. Scores are calculated in real-time and immediately available in the EHR to enhance communication and decision-making. Scores that meet or exceed severity thresholds, or indicate an endorsed need, trigger an ‘alert’ via EHR in-box messaging for clinician intervention. Results from our initial feasibility studies demonstrated the successful EHR-integration and feasible implementation in a single ambulatory cancer care setting [23, 24]. 

### Current work

We conducted a modified stepped wedge trial with a type 2 hybrid effectiveness-implementation design and formally evaluated effectiveness and implementation outcomes from key constituents, including patients [27]. Our implementation efforts were guided by IS models, primarily the RE-AIM (Reach, Effectiveness, Adoption, Implementation, and Maintenance) planning and evaluation framework [28, 29] and the Consolidated Framework for Implementation Research (CFIR) for determinants [30], and we report elsewhere on the use of 34 discrete implementation strategies from the nine categories in the Expert Recommendations for Implementing Change (ERIC) taxonomy [31, 32]. Key implementation strategies included the creation and distribution of education materials (e.g., pamphlets and posters), hosting clinician orientation sessions, and developing EHR smartphrases for easier clinician access to cPRO patient responses. We intentionally provided such ‘light-touch’ guidelines for clinicians to give them agency in how they use cPRO results and approach them with patients.

Here, we examine our efforts to implement cPRO from the patient perspective, focusing on facilitators and barriers they experienced in regularly completing cPRO. Given clinic operational variability, differences across sites and the size and diversity of the patient population, we applied a mixed methods research (MMR) approach, offering us the ability to enhance the depth and quality of data with context to better inform results [33, 34]. 

## Methods

We followed the Standards for Reporting Qualitative Research [35] guidelines for presenting our findings (checklist in Supplementary Material).

### Aim

Our overall aim was to elicit direct feedback from patients regarding their experiences of cPRO implementation during the expansion of cPRO within the NM healthcare system.

### Design

Using an MMR approach, we collected feedback in two waves over one calendar year. We used qualitative methods (*Wave 1*; 1-hour focus groups and individual interviews) to identify themes pertaining to patient acceptability [32], and quantitative methods (*Wave 2*; structured survey of up to 86 items) to conduct a more systematic exploration of identified themes. To include clinics and hospitals in larger urban areas as well as smaller suburban and rural areas, patients from three geographical regions of the Chicago-area NM healthcare system were invited to participate. This project was approved by the Social and Behavioral Research Panel of Northwestern University’s Institutional Review Board (IRB; #STU00207807).

### Setting

We conducted this study at outpatient adult oncology clinics across multiple hospitals within a single healthcare system (NM). Existing regions (Central, North, and West) served as clusters for the larger cluster-randomized stepped wedge trial [33]. The Central region includes a single large, urban-based medical center; the North and West regions are each comprised of smaller hospitals in suburban communities.

### Population

Eligible participants for both waves were 18 years of age or older and met the criteria listed in Table 1.

**Table 1:** Eligibility Criteria

Wave	Eligibility Criteria
*Wave 1* and 2	Diagnosis of an ICD confirmed solid or hematological malignancy
	Received oncology services at an NM location within the previous 12 months
*Wave 1*	Received at least 4 invitations to complete cPRO
	Consent to being audio recorded if participating in a focus group
*Wave 2*	Received at least 2 invitations to complete cPRO
	Completed cPRO at least once
	Did not participate in *Wave 1*

In *Wave 1*, we defined a priori four user groups to ensure representation regarding number of cPRO completions and held separate feedback sessions for each group. User groups were defined as (1) regular users (completed cPRO at least twice), (2) one-time users (completed cPRO once), (3) never users (never completed cPRO), and (4) users who generated clinical alerts (completed cPRO at least twice and responses prompted alert messages to care teams).

### Materials

*Wave 1* feedback sessions were conducted using semi-structured interview guides customized by user group and session type (interview versus focus group). Interview guide content was informed by CFIR [30] to elicit contextual determinants (i.e., barriers and facilitators) from patient perspectives. Questions covered 4 of the 5 CFIR domains [30] (intervention characteristics, outer setting, inner setting, and characteristics of individuals), excluding questions on cPRO’s implementation process as patients would not be privy to process-related determinants. Topics covered participants’ experiences completing cPRO, why they completed it, comprehension of its purpose, and associated care team communications. *Wave 1* sessions were conducted using Zoom Meetings video conference software (Zoom Video Communications Inc., 2016).

*Wave 2* participants completed an online survey based on themes that emerged from *Wave 1* feedback. Survey items addressed patient understanding of cPRO purpose and functionality, care team cPRO use and related clinical communications, exposure to cPRO educational materials, cPRO impact on health management self-efficacy and care, usability, and compliance. Additional items explored the frequency with which participants’ clinicians asked about cPRO symptom domains during clinical encounters. Survey items utilized multiple choice and both 4- and 5-point Likert-type response scales (see Table 4 in Supplementary Material).

### Process

Data collection was sequential with parallel sampling [36] in both waves. Recruitment was purposeful and stratified [36] by region (and by user group for *Wave 1*). We planned to enroll up to 50 patients across regions to provide feedback via focus groups of 6–10 participants for ideal group conditions [37] and, for privacy reasons, individual interviews for those who generated clinical alerts. For *Wave 2*, we aimed to enroll 150 patients (50 from each region) to promote results that yield stable estimates and are representative of our overall population [38].

### Recruitment

We recruited *Wave 1* participants based on eligibility criteria (Table 1), first screening and approaching those from the larger study [39] who consented to re-contact for similar research opportunities, and then those in the NM Enterprise Data Warehouse (an integrated repository of NM clinical data) who had received four or more cPRO invitations as part of their cancer care.

In *Wave 2*, an automated EHR report was used to screen patients cross-regionally. Eligible patients had completed one or more cPRO assessments during the previous 8 calendar months and were recruited via NM EHR patient-portal messaging.

### Data collection

All study participants completed an electronic consent form via the Research Electronic Data Capture (REDCap) platform [40, 41] followed by questions on clinical and sociodemographic characteristics, patient portal usage, and technology use literacy (for descriptive purposes). *Wave 1* participants provided live feedback via video conference. Study team members (including ML, KW, EP) with experience and/or training in qualitative research conducted the sessions, which lasted no longer than 45 min for interviews and 60 min for focus groups. Feedback sessions were audiotaped and transcribed for analysis. Transcripts were de-identified once analysis was complete. For *Wave 2*, we gathered feedback via an electronic survey in REDCap, designed to quantitatively verify endorsement of themes related to barriers and facilitators identified in our *Wave 1* qualitative work. Participants completed 46 to 86 items depending on their responses to items with branching logic.

### Participant compensation

Participants who completed a *Wave 1* session were compensated $50. Participants who completed a *Wave 2* survey were compensated $25.

### Data analysis

Data from *Waves 1* and *2* were cleaned and analyzed with descriptive statistics using IBM SPSS Statistics Version 28.0. Sample characteristics were described with counts and frequencies on variables spanning demographics, health history, and general technology use; analyses were separated by *Wave 1* versus *Wave 2* subgroups.

*Wave 1* focus group data were analyzed directly from session transcripts enabled by Zoom software. Three team members (ML, EP, JC) entered interview responses into an Excel database to enable team coding. We used both rapid and traditional qualitative methods to analyze *Wave 1* data. Team members with experience in qualitative analysis (ML, KW, EP) conducted a rapid analysis [42] of the patient feedback to evaluate initial impressions and inform ongoing cPRO implementation [39, 43, 44]. Given the quality improvement nature of the larger project, analyses focused on identifying facilitators and barriers to successful implementation of PROs in cancer care. We applied a directed content analysis approach using implementation research frameworks [45] identifying, categorizing, and condensing all barriers and facilitators discussed (implicitly or explicitly) by participants until themes emerged. Data were double coded, and the coding team met regularly with a principal investigator (SG) to refine emergent themes and resolve disagreements in coding.

Wave 2 data were described using counts and frequencies for each level of categorical variables. Continuous variables were described with means and standard deviations. A small amount of missing data varied across questions, ranging from 0 (0.0%) to 6 (4.2%). Therefore, we present descriptive statistics using complete cases for each variable, which is acceptable for this degree of missingness [46]. 

## Results

The final analytic sample size was 180 (*n* = 37 from *Wave 1* and *n* = 143 from *Wave 2*). Participants were equally represented across the three regional cancer centers sites. Participants’ mean age was 62.9 years (range 33–90) and mean age of diagnosis was 57.6 years (range 26–85). The majority were female, White, non-Hispanic, and married; represented various solid tumor types and hematologic malignancies (with breast cancer diagnoses being predominant); and were relatively equally distributed by treatment status (Table 2). Our sample reported a high level of education, computer literacy, and patient portal usage. Over three-fourths of participants indicated they were “Very Comfortable” using computers or touchscreen devices and used the patient portal frequently.

**Table 2.** Participating patient characteristics

Characteristic	Wave 1 (n=37)	Wave 2 (n=143)
	n (%)	n (%)
cPRO User Group		
Never User	3 (8.1%)	--
Central	1 (2.7%)	
West	1 (2.7%)	
North	1 (2.7%)	
Regular User	16 (43.2%)	--
Central	6 (16.2%)	
West	5 (13.5%)	
North	5 (13.5%)	
User Generating Clinical Alert(s)	18 (48.6%)	--
Central	6 (16.2%)	
West	6 (16.2%)	
North	6 (16.2%)	
Cross-Cohort	--	143 (100%)
Healthcare System Region		
Central	13 (35.1%)	49 (34.3%)
West	12 (32.4%)	46 (32.2%)
North	12 (32.4%)	48 (33.6%)
Gender Identity		
Female	28 (75.7%)	88 (61.5%)
Male	9 (24.3%)	53 (37.1%)
Not listed/missing	0.0%	2 (1.4%)
Age at recruitment - Mean (Range)	59.56 (33– 86)	63.72 (36– 90)
Age at diagnosis– Mean (Range)	56.77 (26– 83)	57.87 (26– 85)
Ethnicity		
Non-Hispanic	34 (91.9%)	136 (95.8%)
Hispanic	2 (5.4%)	2 (1.4%)
Declined	1 (2.7%)	4 (2.8%)
Race (check all that apply)		
White	35 (94.6%)	139 (97.2%)
Black or African American	0 (0.0%)	0 (0.0%)
Asian	2 (5.4%)	0 (0.0%)
Native Hawaiian or Other Pacific Islander	1 (2.7%)	0 (0.0%)
More than one race	1 (2.7%)	3 (2.1%)
Other	0 (0.0%)	5 (3.5%)
Marital Status		
Single/Never married	6 (16.2%)	6 (4.2%)
Married	25 (67.6%)	109 (76.2%)
In a committed relationship	0 (0.0%)	4 (2.8%)
Separated	0 (0.0%)	1 (0.7%)
Divorced	5 (13.5%)	11 (7.7%)
Widowed	1 (2.7%)	10 (7.0%)
Missing	0.0%	2 (1.4%)
Highest Education		
Less than high school grad.	0 (0.0%)	1 (0.7%)
Some high school	0 (0.0%)	2 (1.4%)
High school graduate	2 (5.4%)	9 (6.3%)
Some college/technical degree/Associates degree	8 (21.6%)	28 (19.6%)
College degree	12 (32.4%)	39 (27.3%)
Advanced degree (MA, MS, MBA, PhD., MD, JD)	15 (40.5%)	63 (44.1%)
Missing	0 (0.0%)	1 (0.7%)
Employment Status		
Full-time employed	10 (27.0%)	57 (39.9%)
Part-time employed	3 (8.1%)	10 (7.0%)
Homemaker	3 (8.1%)	2 (1.4%)
Unemployed	0 (0.0%)	2 (1.4%)
On leave of absence	1 (2.7%)	2 (1.4%)
On disability	3 (8.1%)	6 (4.2%)
Retired	15 (40.5%)	63 (44.1%)
Prefer not to answer	2 (5.4%)	1 (0.7%)
Frequency of MyChart (NM Patient Portal) Use		
Never	0 (0.0%)	0 (0.0%)
Rarely	1 (2.7%)	2 (1.4%)
Sometimes	7 (18.9%)	25 (17.5%)
Often	29 (78.4%)	114 (79.7%)
I don’t have a MyChart account	0 (0%)	0 (0.0%)
Missing	0 (0.0%)	2 (1.4%)
Comfort with Computer/Touch screen device		
Not at all comfortable	0 (0/0%)	0 (0.0%)
A little comfortable	1 (2.7%)	0 (0.0%)
Somewhat comfortable	7 (18.9%)	11 (7.7%)
Very comfortable	29 (78.4%)	130 (90.9%)
I have never used a computer or touchscreen device	0 (0.0%)	0 (0.0%)
Missing	0 (0.0%)	2 (1.4%)
Frequency of Using Computer/Touchscreen device		
Never	0 (0.0%)	0 (0.0%
Monthly	0 (0.0%)	0 (0.0%)
Weekly	0 (0.0%)	5 (3.5%)
Daily	37 (100.0%)	137 (95.8%)
Missing	0 (0.0%)	1 (0.7%)
Type of cancer		
Breast	15 (40.5%)	44 (30.8%)
Bladder	0 (0.0%)	1 (0.7%)
Cervical	0 (0.0%)	3 (2.1%)
Colorectal	2 (5.4%)	10 (7.0%)
Head/neck	0 (0.0%)	3 (2.1%)
Leukemia	0 (0.0%)	15 (10.5%)
Liver	1 (2.7%)	0 (0.0%)
Lung	1 (2.7%)	6 (4.2%)
Lymphoma	4 (10.8%)	18 (12.6%)
Multiple Myeloma	2 (5.4%)	10 (7.0%)
Neuroendocrine	1 (2.7%)	1 (0.7%)
Ovarian	4 (10.8%)	6 (4.2%)
Pancreatic	0 (0.0%)	2 (1.4%)
Prostate	1 (2.7%)	12 (8.4%)
Other	6 (16.2%)	12 (8.4%)
Household Income		
Up to $29,999	2 (5.4%)	4 2.8%)
$30,000 to $59,999	3 (8.1%)	15 (10.5%)
$60,000 to $100,000	13 (35.1%)	29 (20.3%)
Greater than $100,000	13 (35.1%)	77 (53.8%)
Unsure	0 (0.0%)	1 (0.7%)
Prefer not to Answer	5 (13.5%)	17 (11.9%%)
Missing	1 (2.7%)	0 (0.0%)
Stage of cancer		
Stage I	4 (10.8%)	11 (7.7%)
Stage II	8 (21.6%)	8 (5.6%)
Stage III	4 (10.8%)	11 (7.7%)
Stage IV	7 (18.9%)	21 (14.7%)
In remission or cured	9 (24.3%)	51 (35.7%)
Other	2 (5.4%)	13 (9.1%)
Unknown	3 (8.1%)	27 (18.9%)
Missing	0 (0.0%)	1 (0.7%)
Currently receiving cancer treatment		
Yes	15 (40.5%)	78 (54.5%)
No	22 (59.5%)	65 (45.5%)

### Wave 1 results

Although we recruited and collected data separately from distinct user groups, formative review of preliminary findings indicated responses across regions and user groups were highly uniform. Therefore, we report cPRO user group data in a consolidated manner. No new themes emerged after analyzing data from 37 participants, indicating that we had reached saturation [47]. 

The feedback from *Wave 1* sessions fell into four themes: (1) practical facilitators; (2) conceptual facilitators and motivators for regularly completing cPRO; (3) practical barriers; and (4) conceptual barriers to completing cPRO. The study team defined practical barriers as more objective and having a tangible or simple solution, and practical facilitators and motivators as those that directly prompt or enable a specific action. Conceptual barriers, facilitators, and motivators were defined as being more subjective and rooted in participants’ perceptions and understandings of various aspects of cPRO. Exemplary quotes from themes and sub-themes are presented in Table 3.

#### Practical facilitators and motivators

Some participants expressed appreciation for how completing cPRO enabled them to track their own progress and aided communication with their care team outside of appointments. Completing cPRO led some to think about their symptoms ahead of time and feel more prepared for their doctor visits. Others expressed that completing cPRO helped them feel less pressure to remember everything about their symptoms at appointments, which is especially helpful when cognition is affected by cancer treatment. These factors led to what some described as more efficient appointments, which is advantageous when time is limited. Other practical factors included the relative ease of completing cPRO. Participants reported that the EHR-integrated questionnaires were easy to find and complete and that the time required was reasonable. The facilitating practical factor reported most often was acknowledgement of cPRO by their care team (e.g., asking patients to look for cPRO email invitations or referencing participants’ responses during appointments).

#### Conceptual facilitators and motivators

Participants described various examples of cPRO’s value. For example, they appreciated healthcare teams asking how patients are doing, increasing the perception that they care. Some were comforted, feeling that cPRO made their care team more informed. Regularly completing cPRO was also viewed as a way to increase participation in their own care. Participants also viewed cPRO as helpful for reflection; some described how cPRO led to expanding or organizing their thoughts about their symptoms relative to those listed in the questionnaire. They also expressed appreciation for the inclusion of psychological symptoms as an in-depth way to reflect and report on mental health. Most interview participants reported that cPRO questions were relevant to their cancer experiences (due to time constraints, this question was left out of focus groups). When asked what additional symptoms they would add to cPRO, participants offered some ideas, but we found no consistently mentioned symptoms, further suggesting cPRO’s relevancy.

#### Conceptual barriers

While participants found cPRO questions germane to their experiences overall, they also mentioned instances where cPRO did not feel relevant. Some described how certain items did not apply to them, at the current time or before cancer (e.g., ability to do yardwork—a PROMIS item) [48]. Others felt the items did not match with what they wanted their care team to know or the reason for their visit. For example, some said evaluating symptoms would be more useful after a treatment visit rather than before seeing their oncologist. Some also questioned the value of completing symptom monitoring questionnaires after entering post-treatment survivorship.

The other primary conceptual barrier was confusion and uncertainty regarding various aspects of cPRO. One source of confusion was whether to complete cPRO items in reference to cancer-specific experiences only or to consider all factors (like aging). Participants were also unsure of the source and purpose of the questionnaire, who views responses, and how responses are used. Common misconceptions included the questionnaires being used for research or tied to evaluating patient satisfaction, rather than directly informing their care.

#### Practical barriers

Practical barriers reported by participants primarily pertained to (1) specific cPRO items and how they were asked and (2) lack of communication and education from the healthcare system. Participants reported growing tired of answering questions they felt were redundant, particularly post-treatment, and felt they had nothing new to report symptom-wise. Some participants thought the ‘past 7-day’ time frame was too short and that a larger range, like one month, would be more effective, especially in capturing symptoms driven by treatment. Flexibility in how to respond was also desired, for example being able to skip items, write-in additional symptoms, or indicate desire to speak directly with their doctor about certain symptoms. Many participants wanted cPRO to include open text options to provide additional details about their Likert responses.

The other primary practical barrier was lack of acknowledgement from participants’ care teams, including lack of education about cPRO from the healthcare system. All *Wave 1* participants reported seeing no educational materials regarding cPRO (or could not remember seeing any). Some expressed a need for an orientation to cPRO’s purpose and how it is used. While some participants reported various forms of acknowledgement by their care team, most stated that no one had referenced cPRO. Some wondered if their care team used or even looked at the responses. Given the lack of acknowledgment, especially during appointments, some participants came to expect they would have to repeat the same information in appointments that they reported in the cPRO questionnaires. One participant explained how she had diligently completed cPRO but stopped after having to repeat herself during appointments (Table 3).

**Table 3:** Participant Quotes

Domains and Factors	Exemplary Quotes
Practical Facilitators and Motivators	
Track own progress	*“I think it improved my awareness of where I’m at and how I feel. The screener helped me recall what did happen to me at certain times.” (205)*
	*“It makes me think about my own case a little more.” (202)*
Help remember symptoms	*“Cancer patients sometimes forget things in the visit, due to memory issues and the stress of the visit.” (203)*
	*“What I really did like about it what it seemed to cut down on what I had to remember the day of my chemo treatment because I would fill out [cPRO] beforehand.” (NRFG)*
Ease of completion	*“It’s brief and you can do it fast; it’s very straightforward.” (105)*
	*“[cPRO is brief enough and easy enough with the multiple choices that were there, easy to fill out.” (WRFG)*
Care team acknowledgement	*“Usually the nurse asks before [the visit]. I guess they can see my responses and they’ll ask me about some of my responses if I want to follow-up about anything.” (303)*
	*I was contacted by the social worker. I think it was addressed very well. Everyone was very caring and understanding, and made a point that I could reach out if I needed additional assistance.” (205)*
Conceptual Facilitators and Motivators	
Care team more informed	*“I know my oncologist likes to have as much information as they can…I believe it’s helpful to him.” (305)*
	*“As I looked at it, I realized that this is important information for them to know, as it helps you keep an eye on things you might not necessarily mention [in the visit].” (204)*
More involved in own care	*“I keep filling it out because I think I should be doing everything I can to help with my treatment. If it is something that will help with my treatment, then I want to do everything possible to help with that.” (110)*
	*I wanted to…do the best thing that I could for my care.” (210)*
Reflective tool	*“I think [completing cPRO] makes me more aware of where I’m at and how I’m feeling. It helps me recognize when I need to reach out and report an issue.” (205)*
	*A lot of the things you are asked in the screener are things you might not have thought of or been aware of.” (306)*
Conceptual Barriers	
Relevance	*“‘Does your health now limit you doing strenuous activities like hiking, backpacking, etc.’ I haven’t been able to do these activities for 20 years. I can’t jog, I’ve never jogged, but by answering that question, it implies that my cancer contributes to me not being able to do those things.” (401)*
	*“It just kind of left me feeling like, especially as I was got into remission, like don’t even bother filling this out. It just feels repetitious and there’s nothing really new going on, so just kind of felt a little monotonous, I guess, after immediate treatment.” (111)*
Confusion as to purpose of cPRO & how to answer questions	*“It’s hard to separate old age from cancer.” (108)*
	*“Honestly, I wasn’t sure what the screener was. Sometimes, you know, you get questionnaires from different organizations and your responses are compiled somewhere.” (110)*
	*“It’s the hospital sending you a questionnaire about your care.” (306)*
	*“I just do it, so you guys* [researchers] *have it for your notes.” (109)*
Practical Barriers	
Question repetition	*“Some questions seem to be asked numerous times.” (110)*
	*“Are they trying to trick me?” (108)*
Response time range	*“ ‘In the past 7 days’, sometimes I had this dilemma where I had a symptom, but not in that timeframe. I had to answer ‘A Little Bit’ here and there.” (201)*
	*“The ‘In the past 7 days’ time period is difficult for me. In survivorship, changes are better measured in months rather than days.” (107)*
Lack of response flexibility	*“I do like having a section, where you could like type in like a response, it was all picking. It was all like a multiple-choice kind of thing I never had a chance to like type in anything.” (301)*
Lack of care team acknowledgement	*“I sometimes felt like, ‘Is this [cPRO questionnaire] going to anybody?’ It never came up in any of my visits.” (111)*
	*“It would have been nice if the nurse navigator said, ‘Thanks for filling out the questionnaire. Is there anything you want to discuss in more detail/anyone else you’d like to see?’ To follow up and know they got it, because I was doing it in hopes that someone was reading it.” (104)*
	*“I would fill out the screener and then go have a visit with whatever physician I was visiting and then have to repeat myself again in the office, every single thing. So, I didn’t see the value of spending all this time filling out this screener and then having to repeat every single thing. It just feels like doing double the work.” (101)*

**Fig. 1:**
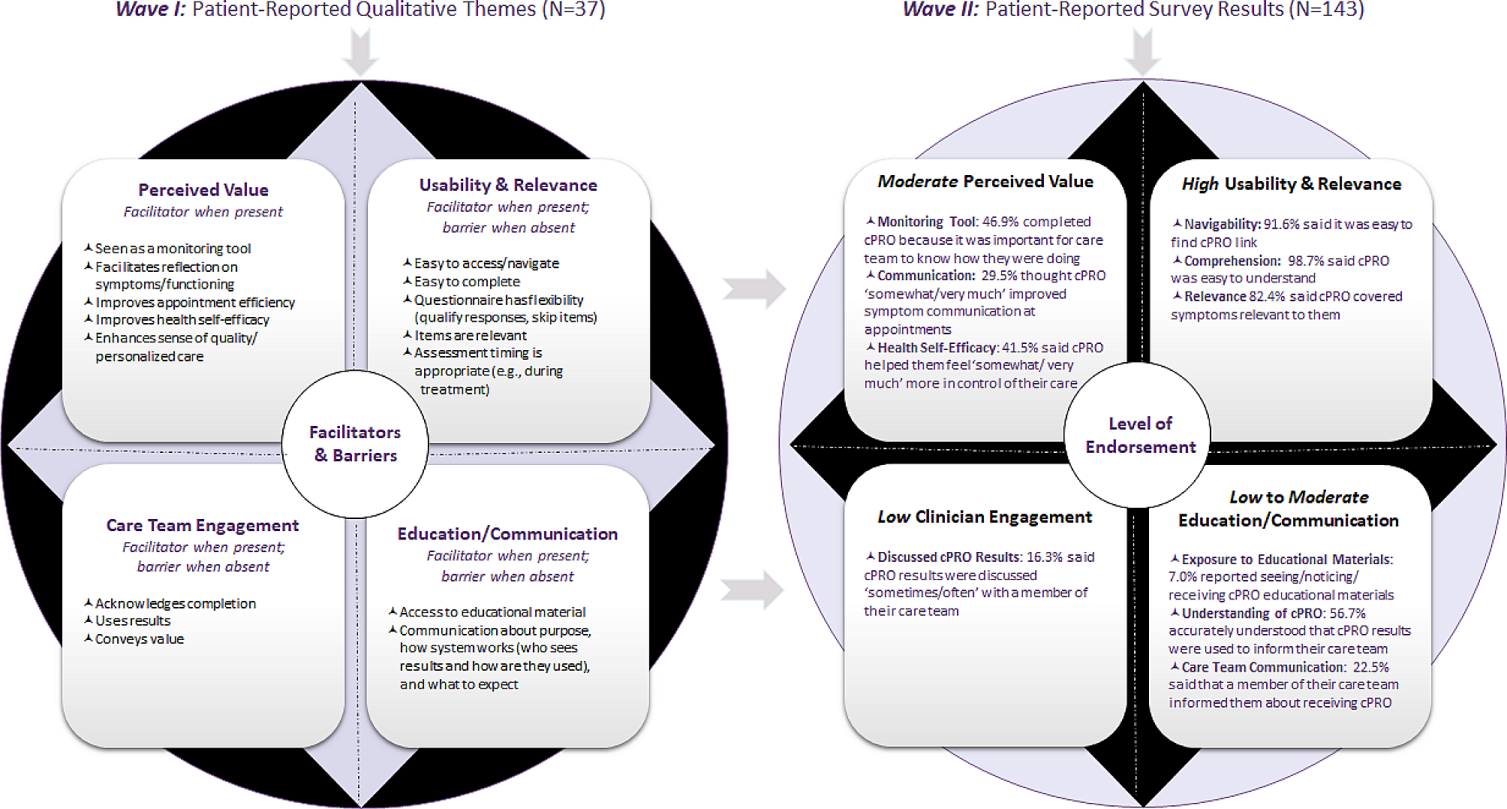
Patient perspectives on cPRO Implementation: Qualitative themes (facilitators and barriers) and survey results (level of endorsement)

In summary, implementation determinants identified by participants broadly relate to the principal domains of *perceived value*, *usability and relevance*, *education and communication*, and *care team engagement*, each of which appears to be a key facilitator when present and a barrier when absent (Fig. 1). Patients saw cPRO’s unique *value* in its ability to monitor symptoms, facilitate reflection, boost self-efficacy, improve appointment efficiency, and strengthen sense of care quality. In terms of *usability and relevance*, patients found cPRO easy to access, navigate and complete and felt items were relevant while desiring additional flexibility when responding. Patients had not seen *educational* materials (brochures and posters) and wanted more *communication* from their care team about cPRO’s purpose and functionality and emphasized the importance of their care team acknowledging their results and referring to completed cPRO rather than asking the same questions again during the visit.

### Wave 2 results

We first asked survey respondents about their general recall of the cPRO screener and most (85.2%) indicated (“Somewhat” to “Very much”) that they remembered completing it. However, when asked about the purpose of cPRO, only just over half (56.7%) accurately understood that cPRO results were used to inform their care team. Others were unsure (13.5%) or thought cPRO was used for research or patient satisfaction assessment (29.0%). Similarly, very few (7.0%) participants reported noticing educational materials about cPRO.

Responses about cPRO usability were unilaterally positive. When asked about navigability, a majority (91.6%) found the questionnaire easy to find (“Somewhat” to “Very much”). In terms of comprehension, there was substantial endorsement (“Somewhat” to “Very much”) that the cPRO questions were easy to understand (98.7%) and easy to answer (98.6%). Participants (82.4%) also indicated (“Somewhat” to “Very much”) that the cPRO questions covered symptoms and needs relevant to them.

We asked respondents why they did or did not complete cPRO. Top reasons for completion included (1) thinking it was important for the care team to know how they were doing (46.9%), (2) being asked to complete it by a care team member (30.8%), and (3) feeling it would improve communication about symptoms and needs with their care team (30.0%). Further, 22.4% of patients thought it would improve the quality of their care. When asked to choose a top reason for completing cPRO, being asked to complete it by a member of their care team was most frequently endorsed (27.1%). While more than half (55.9%) said they complete cPRO whenever they are asked, the top reason for non-completion was lack of time (16.1%).

Cancer care team communication about cPRO was reported as lower than anticipated; only some patients (22.5%) indicated with confidence that a member of their care team discussed cPRO. Many more said they were unsure (39.4%) or their care team never mentioned cPRO (38.0%). Further evidence of low clinician-to-patient communication about cPRO was evidenced in a 16.3% endorsement of how often cPRO results were discussed with a member of their care team (“Sometimes” or “Often”). Interestingly, despite reporting low care-team engagement, almost a third (29.5%) of participants felt (“Somewhat” to “Very much”) that completing cPRO had improved communication about their symptoms and needs with their care team. Similarly, 41.5% reported (“Somewhat” to “Very much”) that completing cPRO helped them feel more in-control of their care.

Finally, we asked patients to rate, according to their general experience, how often their doctor asks them about the five symptom domains included in cPRO. Although not a direct assessment of a patient-facing implementation determinant, we aimed to better understand the perceived frequency with which these symptoms are addressed during routine care, independent of the cPRO assessment (to provide contextual information). A relatively high percentage of participants reported that clinicians “Sometimes” or “Often” ask about fatigue/tiredness (72.3%), pain interference (69.5%), and physical functioning (67.1%) during appointments. Consistent with other research findings, fewer participants reported routine inquiry (“Sometimes” or “Often”) about mental health concerns (50.3% for worry/anxiety and 43.2% for sadness/low mood) [8, 49–51]. 

Results from the Wave 2 survey helped us understand the degree to which identified facilitators and barriers were endorsed or experienced by patients (see Fig. 1). Broadly, results suggest *high* (82–99%) endorsement of usability and relevance (items are relevant and easy to comprehend; the system is navigable), *moderate* (30–47%) endorsement of perceived value (cPRO improves communication at appointments and sense of self-efficacy; useful as a monitoring tool), *low to moderate* (7–57%) endorsement of education and communication (saw educational materials; care team communicated about cPRO; understood purpose of cPRO) and *low* (16%) endorsement of care team engagement (care team acknowledged/discussed cPRO results).

### Initial impact

Rapid analysis of this mixed methods data set has already led to effective, measurable improvements in cPRO implementation. Upon presenting preliminary findings to the implementation team, they responded to patient frustration over cPRO item redundancy by designing a shorter version of the tool (moving from PROMIS computer adaptive tests to two-item short form measures but still assessing the same domains), which also reduced average completion time from 6 to 7 to 2–3 min. This change significantly improved completion rates (increases of 9–35% over 18 months), also addressing the *Wave 2* finding that showed lack of time as the top reason patients do not complete cPRO. Additionally, the study team clarified within the assessment instructions that patients should answer questions based on symptoms due to any cause, addressing patients’ uncertainty about responding to symptoms or needs that are driven by factors other than cancer. Finally, in response to participants’ desire for more feedback from their clinical care team about their cPRO responses, we added a mechanism into the EHR informing patients whether their clinicians saw their results (via a smartphrase incorporated into progress notes & visible in patients’ after-visit summaries).

## Discussion

This mixed methods analysis explored patient perspectives of healthcare system-wide implementation of cPRO, an electronic health record-integrated patient-reported symptom and needs monitoring program, as part of routine cancer care. After conducting semi-structured discussions with patients in *Wave 1*, we sought to confirm and expand on emergent themes via a survey completed by a larger sample in *Wave 2*. Collectively, these data provided insight on patient attitudes and experiences that can inform actionable changes to cPRO implementation. Results centered on four principal domains that appear to enhance or detract from patient uptake and adherence and point to implementation strategy enhancements needed to improve reach, adoption, sustainability, and effectiveness.

Findings aligned with what we had learned anecdotally from clinicians, administrators, and patients during cPRO implementation, but there were some unexpected results that contribute to the literature on facilitators and barriers to implementing electronic patient-reported symptom monitoring programs.

First, patients found value in cPRO, including that it improved communication with their care team, despite low care team engagement. Likewise, a significant number of patients (42%) indicated (“Somewhat” to “Very much”) that cPRO enhanced their sense of self-efficacy, a desirable patient-centered benefit, pointing to how symptom monitoring programs like cPRO can activate patients [52]. Specifically, patients described how cPRO facilitated thoughtful reflection on their symptoms and needs and better prepared them to communicate concerns in medical visits. This finding maps onto one of the basic principles of patient-clinician communication: “the right information,” (i.e., patients sharing relevant symptoms and experiences) [53]. Finally, it is noteworthy that a quarter to over half (27.7-56.7%) of survey respondents said they were “Never” or “Rarely” asked, within routine care, about some of the most common physical and, especially, psychological symptoms reported in oncology settings, This finding highlights the general need for symptom monitoring, and the specific need for mental health surveillance in cancer care [54]. 

Analysis of these data have prompted effective changes to cPRO implementation, including designing a shorter version of the tool, which led to increased completion rates. The investigators will continue to use these findings to guide additional implementation strategies focused on enhancing communication, education, and clinician engagement. Because participants reported not remembering educational materials, we plan to increase and expand educational materials to help them understand the value of regular cPRO completion. Doing so is more feasible now that handouts and other materials are allowed in clinics again, following previous COVID-19 restrictions limiting widespread distribution during our data collection period. We are also working to create a patient-facing video, providing care teams with cPRO talking points, and tracking distribution of patient education materials.

Further, our results suggest that enhancing clinician communication and engagement via more intensive clinician-facing implementation strategies may improve compliance and patients’ cPRO-related experiences. Topics to explore include: what motivates clinicians to use cPRO results and discuss them with patients; how often clinicians discuss different symptoms during visits; how they choose what to discuss; how these discussions affect care; how the degree of clinician acknowledgement motivates patients; and whether cPRO should be addressed at all visits or more selectively.

### Limitations and strengths

The time between most recent cPRO completion and feedback session participation varied across participants, which potentially impacted recall. We are also unable to generalize our findings to the entire local cancer population because most participants regularly used cPRO and had high technology literacy. Our study participants’ demographics and our implementation of cPRO in a well-resourced academic health center limits generalizability to more diverse populations and other settings [55]. In particular, this study’s sample was less diverse in terms of race and ethnicity, including compared to larger efficacy analysis data set for the parent study, which recruited from the same clinics—limiting the generalizability of findings. Additionally, most data collection occurred during various phases of the COVID-19 pandemic, when in-clinic appointments were minimal and physicians were overburdened. As a result, in-clinic cPRO administration was largely not feasible, and we were reluctant to use higher-touch implementation strategies that demanded greater clinician effort. Finally, the finding that patients reported low frequency of clinicians discussing cPRO results needs to be examined further. Future work should examine what prompts clinicians to discuss results (e.g., severe symptoms or worsening).This work also has various strengths. We explored patient perspectives on an EHR-integrated symptom and needs monitoring program as it was being implemented into standard care across a large academic healthcare system. By purposefully sampling patients who had engaged with cPRO to different extents, our results capture different perspectives regarding EHR-integrated symptom monitoring. Using a mixed methods approach guided by implementation frameworks, we amplified patient perspectives, which are not often highlighted within implementation processes. Further, we demonstrate how this kind of assessment (including rapid analysis), conducted while implementation was underway, can inform program improvements. That approach facilitated iterative changes to cPRO and will inform future versions that reflect patient preferences and experiences.

## Conclusion

Adult oncology outpatients found completing cPRO easy to do and valuable, but they also were confused about key aspects of the tool and emphasized the importance of education and clinician engagement to motivate sustained regular completion. Their feedback offers important insight to inform actionable changes. Informed by these data, future cPRO implementation strategies and modifications should target increasing clinician engagement and patient education to further enhance perceived value, compliance, and, ultimately, higher quality, patient-centered cancer care.

### Electronic supplementary material

Below is the link to the electronic supplementary material.

Supplementary Material 1

Supplementary Material 2

## Data Availability

The datasets generated and/or analyzed during the current study are not yet publicly available but are available from the corresponding author on reasonable request. Once the trial has closed and its results disseminated, anonymized data supporting the conclusions of this, and other published articles will be shared in a publicly accessible way with the research community at large.

## Abbreviations

CFIR: Consolidated Framework for Implementation Research
cPRO: Cancer Patient-Reported Outcomes
EHR: Electronic health record
IS: Implementation Science
IRB: Institutional Review Board
MMR: Mixed Methods Research
NM: Northwestern Medicine
PRO: Patient-reported outcomes
QOL: Quality of life
REDCap: Research Electronic Data Capture
