# Supplementary material for: A mixed methods evaluation of patient perspectives on the implementation of an electronic health record-integrated patient-reported symptom and needs monitoring program in cancer care
Source: J Patient Rep Outcomes. 2024 Jul 2;8:66. doi: 10.1186/s41687-024-00742-8 (PMC11219691; doi:10.1186/s41687-024-00742-8)
Supplement: Supplementary file 2 — Supplementary Material 2 [file 41687_2024_742_MOESM2_ESM.docx]

Supplementary Table 4. Wave 2 cPRO Use Questions (N=143)

| **Question and Responses** | **N (%) or M (SD)** |
| --- | --- |
| Total number of cPRO completions (n = 141) |  |
| 1 time only | 7 (5.0%) |
| 2-4 times | 57 (40.4%) |
| 5 times or more | 77 (54.6%) |
|  |  |
| cPRO completion method (check all that apply) (n = 143) |  |
| Prior to my appointment, using the MyNM (MyChart) website | 120 (83.9%) |
| Prior to my appointment, using the MyNM mobile application | 35 (24.5%) |
| In-clinic, using a tablet provided by clinical staff | 5 (3.5%) |
| In-clinic, using my own tablet or mobile device | 1 (0.7%) |
| In-clinic, handwriting my responses on paper/laminate | 13 (9.1%) |
| In-clinic, using the computer workstation provided by clinical staff | 0 (0.0%) |
| Over-the-phone, with clinic staff support | 3 (2.1%) |
|  |  |
| In general, how well do you remember completing the cPRO questionnaire? (n = 142) |  |
| 0 - Not at all | 6 (4.2%) |
| 1 – A little bit | 15 (10.6%) |
| 2 - Somewhat | 52 (36.6%) |
| 3 – Quite a bit | 42 (29.6%) |
| 4 – Very much | 27 (19.0%) |
| *Mean (SD)* | *2.49 (1.05)* |
|  |  |
| Understanding of what happens with cPRO responses (n = 141) |  |
| Used to report feedback/satisfaction about medical care | 27 (19.1%) |
| Used as data for research | 14 (9.9%) |
| Used to inform care team about how I’m doing | 80 (56.7%) |
| Other | 1 (0.7%) |
| Unsure | 19 (13.5%) |
|  |  |
| Preferences for how cPRO responses should be used (check all that apply) (n = 143) |  |
| Someone on my care team looks at the answers | 50 (35.0%) |
| The care team determines whether response or help is needed, depending on the results | 74 (51.7%) |
| My doctor mentions it/discusses it with me | 41 (28.7%) |
| Someone other than my doctor reaches out/discusses it with me | 14 (9.8%) |
| Nothing | 14 (9.8%) |
| Unsure | 17 (11.9%) |
| Other | 4 (2.8%) |
|  |  |
| Prior exposure to educational materials about cPRO (n = 142) |  |
| Yes | 10 (7.0%) |
| No | 99 (69.7%) |
| Unsure | 33 (23.2%) |
|  |  |
| Did a member of your care team inform you that you would be receiving cPRO questionnaires as part of your care at NM? (n = 142) |  |
| Yes | 32 (22.5%) |
| No | 54 (38.0%) |
| Unsure | 56 (39.4%) |
|  |  |
| If yes, which care team member informed you? (n = 32) |  |
| Oncologist | 6 (18.8%) |
| An Advanced Practice Provider | 10 (31.3%) |
| Nurse | 10 (31.3%) |
| Social Worker | 1 (3.1%) |
| Don’t remember | 5 (15.6%) |
|  |  |
| Frequency of discussing cPRO with care team (n = 141) |  |
| Never | 76 (53.9%) |
| Rarely | 26 (18.4%) |
| Sometimes | 16 (11.3%) |
| Often | 7 (5.0%) |
| Unsure | 16 (11.3%) |
|  |  |
| Care team member who discussed cPRO results (check all that apply) (n = 143) |  |
| My doctor | 17 (11.9%) |
| Advanced Practice Provider | 13 (9.1%) |
| Nurse | 13 (9.1%) |
| Social Worker | 4 (2.8%) |
| Dietician | 0 (0.0%) |
| Other | 0 (0.0%) |
| Don’t remember | 16 (11.2%) |
|  |  |
| Frequency doctor asks about fatigue/tiredness (n = 141) |  |
| 1 - Never | 10 (7.1%) |
| 2 - Rarely | 29 (20.6%) |
| 3 - Sometimes | 35 (24.8%) |
| 4 - Often | 67 (47.5%) |
| *Mean (SD)* | 2.13 (0.98) |
|  |  |
| Frequency doctor asks about pain that interferes with day-to-day activities (n = 141) |  |
| 1 - Never | 27 (19.1%) |
| 2 - Rarely | 16 (11.3%) |
| 3 - Sometimes | 30 (21.3%) |
| 4 - Often | 68 (48.2%) |
| *Mean (SD)* | 1.99 (1.17) |
|  |  |
| Frequency doctor asks about ability to perform activities of daily living (n = 140) |  |
| 1 - Never | 28 (20.0%) |
| 2 - Rarely | 18 ((12.9%) |
| 3 - Sometimes | 38 (27.1%) |
| 4 - Often | 56 (40.0%) |
| *Mean (SD)* | 1.87 (1.15) |
|  |  |
| Frequency doctor asks about worry or anxiety (n = 141) |  |
| 1 - Never | 31 (22.0%) |
| 2 - Rarely | 39 (27.7%) |
| 3 - Sometimes | 34 (24.1%) |
| 4 - Often | 37 (26.2%) |
| *Mean (SD)* | 1.55 (1.11) |
|  |  |
| Frequency doctor asks about sadness or low mood (n = 141) |  |
| 1 - Never | 43 (30.5%) |
| 2 - Rarely | 37 (26.2%) |
| 3 - Sometimes | 25 (17.7%) |
| 4 - Often | 36 (25.5%) |
| *Mean (SD)* | 1.38 (1.17) |
|  |  |
| I understand why I am asked to complete cPRO (n = 142) |  |
| 1 - Not at all | 3 (2.1%) |
| 2 - A little bit | 11 (7.7%) |
| 3 - Somewhat | 51 (35.9%) |
| 4 - Quite a bit | 46 (32.4%) |
| 5 - Very much | 31 (21.8%) |
| *Mean (SD)* | 2.64 (0.98) |
|  |  |
| cPRO questionnaire is easy to find in MyChart (n = 142) |  |
| 1 - Not at all | 3 (2.1%) |
| 2 - A little bit | 9 (6.3%) |
| 3 - Somewhat | 44 (31.0%) |
| 4 - Quite a bit | 38 (26.8%) |
| 5 - Very much | 48 (33.8%) |
| *Mean (SD)* | 2.84 (1.04) |
|  |  |
| cPRO questions are easy to understand (n = 142) |  |
| 1 - Not at all | 0 (0.0%) |
| 2 - A little bit | 2 (1.4%) |
| 3 - Somewhat | 12 (8.5%) |
| 4 - Quite a bit | 64 (45.1%) |
| 5 - Very much | 64 (45.1%) |
| *Mean (SD)* | 3.34 (0.69) |
|  |  |
| cPRO questions are easy to answer (n = 141) |  |
| 1 - Not at all | 1 (0.7%) |
| 2 - A little bit | 1 (0.7%) |
| 3 - Somewhat | 20 (14.2%) |
| 4 - Quite a bit | 64 (45.4%) |
| 5 - Very much | 55 (39.0%) |
| *Mean (SD)* | 3.21 (0.76) |
|  |  |
| cPRO questionnaire covers symptoms and needs that are relevant to me (n = 142) |  |
| 1 - Not at all | 7 (4.9%) |
| 2 - A little bit | 18 (12.7%) |
| 3 - Somewhat | 50 (35.2%) |
| 4 - Quite a bit | 46 (32.4%) |
| 5 - Very much | 21 (14.8%) |
| *Mean (SD)* | 2.39 (1.05) |
|  |  |
| My care team used the results from my cPRO questionnaire (n = 141) |  |
| Not at all | 19 (13.5%) |
| A little bit | 8 (5.7%) |
| Somewhat | 17 (12.1%) |
| Quite a bit | 9 (6.4%) |
| Very much | 7 (5.0%) |
| Unsure | 81 (57.4%) |
|  |  |
| Completing cPRO questionnaires has improved communication about my symptoms and needs with my care team. (n = 142) |  |
| Not at all | 35 (24.6%) |
| A little bit | 14 (9.9%) |
| Somewhat | 24 (16.9%) |
| Quite a bit | 10 (7.0%) |
| Very much | 8 (5.6%) |
| Unsure | 51 (35.9%) |
|  |  |
| Completing cPRO questionnaires makes me feel more in control of my care (n = 142) |  |
| Not at all | 49 (34.5%) |
| A little bit | 17 (12.0%) |
| Somewhat | 33 (23.2%) |
| Quite a bit | 15 (10.6%) |
| Very much | 11 (7.7%) |
| Unsure | 17 (12.0%) |
|  |  |
| Reasons for completing cPRO (check all that apply) (n = 143) |  |
| A member of my care team asked me to complete it | 44 (30.8%) |
| I thought it was important for my care team to know how I’m doing/feeling | 67 (46.9%) |
| I thought it would improve the quality of my care | 32 (22.4%) |
| I thought it would improve communication about my symptoms and needs with my care team | 54 (37.8%) |
| I have received additional support from my care team in the past based on my responses | 5 (3.5%) |
| I find value in completing the questionnaire and seeing how I’m doing | 23 (16.1%) |
| The questionnaire asks about symptoms I may otherwise not be comfortable discussing in person | 13 (9.1%) |
| Other | 17 (11.9%) |
|  |  |
| Top reason for completing cPRO (select only one) (n = 140) |  |
| A member of my care team asked me to complete it | 38 (27.1%) |
| I thought it was important for my care team to know how I’m doing/feeling | 35 (25.0%) |
| I thought it would improve the quality of my care | 9 (6.4%) |
| I thought it would improve communication about my symptoms and needs with my care team | 23 (16.4%) |
| I have received additional support from my care team in the past based on my responses | 2 (1.4%) |
| I find value in completing the questionnaire and seeing how I’m doing | 14 (10.0%) |
| The questionnaire asks about symptoms I may otherwise not be comfortable discussing in person | 5 (3.6%) |
| Other | 14 (10.0%) |
|  |  |
| Reasons for not completing cPRO (check all that apply) (n = 143) |  |
| Not applicable, I completed the questionnaire whenever I was asked | 80 (55.9%) |
| I didn’t have time | 23 (16.1%) |
| I didn’t feel well | 5 (3.5%) |
| I didn’t understand why I was being asked to complete the questionnaire | 5 (3.5%) |
| I didn’t think it was important | 7 (4.9%) |
| The questionnaires are not relevant to me | 8 (5.6%) |
| The questions are emotionally upsetting | 2 (1.4%) |
| The questionnaire format is too restrictive | 10 (7.0%) |
| The questionnaire takes too much time to complete | 3 (2.1%) |
| I am asked to complete the questionnaires too frequently | 12 (8.4%) |
| The questionnaire format is too impersonal | 6 (4.2%) |
| Other | 16 (11.2%) |
|  |  |
| Top reason for not completing cPRO (select only one) (n = 137) |  |
| Not applicable, I completed the questionnaire whenever I was asked | 80 (58.4%) |
| I didn’t have time | 14 (10.2%) |
| I didn’t feel well | 3 (2.2%) |
| I didn’t understand why I was being asked to complete the questionnaire | 1 (0.7%) |
| I didn’t think it was important | 4 (2.9%) |
| The questionnaires are not relevant to me | 7 (5.1%) |
| The questions are emotionally upsetting | 2 (1.5%) |
| The questionnaire format is too restrictive | 6 (4.4%) |
| The questionnaire takes too much time to complete | 1 (0.7%) |
| I am asked to complete the questionnaires too frequently | 5 (3.6%) |
| The questionnaire format is too impersonal | 3 (2.2%) |
| Other | 11 (8.0%) |

**Note.** 143 participants responded to the Wave 2 survey. There was a small amount of missing data that varied across questions from 0 (0.0%) to 6 (4.2%). The table displays the number of respondents for each question and descriptive analyses excluded missing cases at the item level.
